# Effect of ABO blood group on postoperative overall survival and recurrence-free survival rate in patients with hepatocellular carcinoma after hepatectomy: a multi-center retrospective cohort study

**DOI:** 10.1186/s12893-023-02236-8

**Published:** 2023-10-24

**Authors:** Mansour Bahardoust, Maryam Zolfaghari Dehkharghani, Pouya Ebrahimi, Maryam Najafirashed, Safa Mousavi, Meisam Haghmoradi, Mohsen Khaleghian, Adnan Tizmaghz

**Affiliations:** 1 Department of Epidemiology, School of Public Health, Shahid Beheshti University of Medical Sciences, Tehran, Iran; 2 School of Public Health, Department of Health Care Administration and Policy, University of Nevada Las Vegas(UNLV), Las Vegas, NV USA; 3 Ahvaz, Jundishapur University of Medical Sciences, Ahvaz, Iran; 4 School of Medicine, Iran University of Medical Sciences, Tehran, Iran; 5 Department of Public Health, College of Health and Human Services, California State University, Fresno, CA USA; 6 Department of Orthopedic Surgery, Urmia University of Medical Sciences, Urmia, Iran; 7 Vascular Surgery Department of General Surgery, School of Medicine, Iran University of Medical Sciences, Tehran, Iran; 8 Department of General Surgery, School of Medicine, Iran University of Medical Sciences(IUMS), Tehran, Iran

**Keywords:** Hepatocellular carcinoma, Overall survival rate, Recurrence-free survival, ABO blood group

## Abstract

**Background:**

Hepatocellular carcinoma (HCC) is one of the most common malignancies worldwide. The survival rate after hepatectomy as the first line of treatment for HCC depends on various factors. This study evaluated the association of the ABO blood group and Rh with overall survival (OS) and Recurrence-free survival (RFS) rate after hepatectomy.

**Methods:**

This multicenter retrospective cohort study reviewed the medical files of 639 HCC patients who underwent hepatectomy from 2010 to 2022 in three medical centers affiliated with the Iran University of Medical Sciences. Patient data, including demographic, clinical, tumor characteristics, and post-surgery outcomes, were collected by referring to the patient’s medical profiles. The Cox proportional hazard investigated the relationship between ABO blood group type and OS and RFS rate after hepatectomy.

**Results:**

The five-year OS and RFS rates were 25.4% and 18.7%, respectively. The five-year OS (Lok rank:40.89, P:0.001) and RFS rate in patients with blood type A were significantly lower than in non-A patients. (Lok rank:10.8, P:0.001) The multivariate Cox analysis showed that blood type A, age < 45 years, tumor size > 5 cm, Poor tumor differentiation, presence of metastasis, The number of involved lymph nodes ≤ 2, and serum Alpha-Fetoprotein)AFP( level ≥ 400 were significantly related to the decreased survival rate of HCC patients after hepatectomy (*P* < 0.05) There was no significant association between Rh with OS and RFS (*P* > 0.05).

**Conclusion:**

Blood group type A, compared to non-A, can be associated with decreased OS and RFS rates in patients with HCC after hepatectomy.

## Introduction

Hepatocellular carcinoma (HCC) is one of the most common malignancies worldwide. According to the International Agency for Research on Cancer (IARC), it is estimated that the annual number of new cases and deaths from liver cancer by 2040 will be more than 55% increase [1]. The prevalence of HCC is increasing in Asian and African countries and Western countries [2, 3].

HCC is one of the most important and intensifying global health challenges, especially in developing countries [4]. HCC is more common in men than women [5]. Hepatitis B is the most common cause of HCC [5, 6]. HCC, With a 5-year survival rate of 18%, is the third leading cause of cancer death worldwide, indicating the high lethality of this cancer [3, 7]. According to various studies, the 5-year recurrence rate of HCC has been reported between 4.9% and 39.9% [8], indicating that treating this cancer requires a multidisciplinary approach [8].

Hepatectomy, as the first line of treatment for HCC in patients with preserved liver function, is the best treatment method compared to local ablation and chemoembolization [9–12]. However, the survival rate of these patients is still short and unsatisfactory due to the high recurrence of HCC after hepatectomy [13–15]. The survival and recurrence rate of HCC after hepatectomy depends on factors such as tumor stage, maximum tumor diameter, degree of tumor differentiation and, GGT levels [16], histological grade of metastasis, age of patients, body mass index, and alpha-fetoprotein (AFP) levels [17–22].

Recent studies have shown the relationship between the ABO blood group type and the survival rate of some cancers [23–25]. Many studies reported that the ABO blood group type is associated with HCC survival after hepatectomy [22, 26]. While A. Oral et al., did not report a significant relationship between the type of blood group with the survival rate of HCC after hepatectomy [27]. The distribution of blood groups in different countries is different [28].

Based on our knowledge, limited studies have investigated the association of blood group type with overall survival and recurrence-free survival rate of HCC patients after hepatectomy. Considering the subject’s importance [26, 27], this retrospective cohort study aimed to evaluate the effect of the ABO blood group and RH factor on OS and RFS rates after Hepatectomy.

## Methods

### Patient characteristics

The Iran University of Medical Sciences ethics committee approved the present study with the code(IR.IUMS.REC.1401.925). In this multicenter retrospective cohort study, the medical profiles of 956 patients with HCC who underwent resection hepatectomy between 2010 and 2022 in hospitals affiliated with Iran Medical Sciences were reviewed. Six hundred forty-one patients were included. The definitive diagnosis of HCC was made based on pathology and biopsy findings and with an oncologist’s opinion.

Inclusion criteria included patients with a definite diagnosis of HCC and follow-up of at least six months after surgery. Secondary HCC (cases that did not originate from the liver), patients who underwent lobectomy patient records, simultaneous suffering from other cancers, and lack of access to the blood bank were defined as the exclusion criterion.

#### Data collection

Patient data were collected using a three-part checklist, including demographic information, clinical and laboratory findings, tumor characteristics, and post-surgery outcomes by the researcher by referring to the medical profiles. Demographic characteristics included age, sex, education level, Body mass index (BMI), family history of HCC, alcohol consumption, and smoking. The laboratory and clinical information of the patients (hepatitis, hepatitis type, ALP level (ng/ml), Child–Pugh stage, ABO blood group e, and RH factor were. Tumor characteristics included tumor size, number of lymph nodes, cancer stage, tumor differentiation, and metastasis, which were collected by referring to the pathological findings of the patients. The outcomes investigated in this study included the duration of the follow-up, the median survival, the five-year OS, and the RFS rates based on the type of blood group.

Tumor characteristics were classified according to the AJCC 7th Edition Staging System for HCC guidelines [29]. Tumor-node-metastasis index (TNM) classification in this study includes tumor size (< 5 cm/ > 5 cm), number of involved lymph nodes (< 2 and ≥ 2), and presence of metastasis (positive/negative). Survival was defined as the time interval between hepatectomy and death.

### Statistical analysis

Stata 17 and SPSS version 22 software were used for data analysis. Mean ± standard deviation was used to report quantitative variables. Qualitative variables were reported with frequency and (%). Kaplan–Meier was used to estimate patients’ OS and RFS rates after hepatectomy based on tumor characteristics and ABO blood group type. The significance of the Kaplan–Meier estimate for patient survival rate was reported using the Log Rank test. Univariate Cox proportional hazard ratio (HR) analysis was used to investigate the relationship between demographic, clinical, and tumor characteristics of OS and RFS rate after hepatectomy. Multivariate Cox regression analysis.

was used to control the effect of confounding variables. To estimate the adjusted effect of the ABO blood group type, the variables with *P* value < 0.2 [30] in univariate analysis were entered into the analysis of multivariate analysis with the backward method. The effect size with HR adjusted was reported in the 95% confidence interval (95% CI). A p-value less than 0.05 was considered a statistical significance level.

## Results

### Demographic and clinical characteristics

The mean age was 59.18 ± 11.38 years (range 25 to 86 years). The mean BMI was 22.7 ± 2.89 kg/m2. The majority of patients were married. The median survival was 32.5 (interquartile range (IQR): 27.4 to 37.6)months. The mean follow-up was 22.3 ± 56.7 months. The frequency of blood group types O, A, B, and AB was 219(34.2%), 201(31.4%), 148(23.1%), and 73(11.3%), respectively. Demographic and tumor characteristics were compared among four types of blood groups. Most of the patients in all four types of blood groups were 45 to 65 years old. (P: 0.65) The frequency of patients in stage I tumors was 15.4%, 18.3%, 18.2%, and 24.7% for blood groups A, O, B, and AB, respectively. (p:0.089) Nearly half of the patients in all blood groups had tumor size < 5 cm. (p: 16). The frequency of patients with serum AFP levels ≥ 400 ng/ml in patients with blood groups A, O, B, and AB was 33.8%, 34.2%, 33.8 and 14.6% respectively. (P: 0.095) There was no significant difference between the demographic, pathological findings, and tumor characteristics in patients with A and non-A blood groups (Table 1).

**Table 1:** Comparison of demographic and tumor characteristics based on the ABO blood group type

Demographic characteristics	N: 641 patients with HCC	ABO blood group type					ABO blood group type		
		**O** **(N:219)**	**A** **(N:201)**	**B** **(N:148)**	**AB** **(N:73)**	***P* value**	**A** **(N:201)**	**Non-A** **(N:440)**	***P* value**
Age group						0.65			0.54
• < 45 year	211 (32.9%)	69 (31.5%)	65 (32.3%)	54 (36.4%)	23 (29.5%)		65 (32.3%)	146 (33.2%)	
• 45–65 year	312 (48.7%)	107 (48.9%)	104 (51.7%)	74 (50%)	33 (42.3%)		104 (51.7%)	208 (47.3%)	
• > 65 year	118 (18.4%)	43 (19.6%)	32 (16%)	20 (13.6%)	22 (28.2%)		32 (16%)	86 (19.7%)	
BMI (Kg/m^2^)	22.7 ± 2.89	22.9 ± 2.19	23.25 ± 3.11	21.54 ± 2.58	23.8 ± 2.55	0.44	23.25 ± 3.11	22.51 ± 3.09	0.41
Median survival (IQR 1, 3) Month)	32.5 (27.4, 37.6)	33.4 (28.5,38)	29.55 (24.7,34.3)	32.5 (28.1, 36.9)	32.8 (27.6, 37.8)	0.68	29.55 (24.7,34.3)	34.1 (29.4, 38.6)	0.54
Sex						0.88			0.75
• Male	486 (75.8%)	162 (74%)	159 (79.1%)	109 (73.6%)	56 (76.7%)		159 (79.1%)	327 (74. 3%)	
• Female	155 (24.2%)	57 (26%)	42 (20.1%)	39 (26.4%)	22 (23.3%)		42 (20.1%)	113 (25.7%)	
Smoking status						0.11			0.28
• Yes	277 (43.2%)	99 (45.2%)	90 (44.8%)	60 (40.5%)	28 (38.4%)		90 (44.8%)	187 (42.5%)	
• NO	364 (56.8%)	120 (54.8%)	111 (55.2%)	88 (59.5%)	45 (61.6%)		111 (55.2%)	253 (57.5%)	
Family history of GC						0.37			0.45
• Yes	58 (9%)	21 (9.6%)	17 (8.5%)	11 (7.4%)	9 (12.3%)		17 (8.5%)	41 9.3%)	
• No	583 (91%)	198 (90.4%)	184 (91.5%)	137 (92.6%)	64 (87.7%)		184 (91.5%)	399 (90.7%)	
Child–Pugh stage						0.22			0.28
• A	512 (79.9%)	178 (81.3%)	153 (76.1%)	118 (79.7%)	63 (86.4%)		153 (76.1%)	359 (81.6%)	
• B	62 (9.7%)	25 (11.4%)	21 (10.5%)	11 (7.4%)	5 (6.8%)		21 (10.5%)	41 (9.3%)	
• Unknown & missing	67 (10.4%)	16 (7.3%)	27 (13.4%)	19 (12.9%)	5 (6.8%)		27 (13.4%)	40 (9.1%)	
Tumor size (cm)						0.16			0.19
• ≤ 5	317 (49.5%)	105 (47.9%)	93 (46.3%)	71 (48%)	43 (59%)		93 (46.3%)	224 (51%)	
• > 5	236 (36.8%)	82 (37.4%)	79 (39.3%)	50 (33.8%)	25 (34.2%)		79 (39.3%)	157 (35.7%)	
• Unknown & missing	88 (13.7%)	32 (14.7%)	29 (14.4%)	22 (18.2%)	5 (6.8%)		29 (14.4%)	59 (13.3%)	
Metastasis						0.088			0.11
• Yes	328 (51.2%)	104 (47.5%)	100 (49.8%)	79 (53.4%)	43 (58.9%)		100 (49.8%)	228 (51.8%)	
• No	110 (17.1%)	40 (18.2%)	36 (17.9%)	31 (20.9%)	5 (6.8%)		36 (17.9%)	74 16.8%)	
• Unknown & missing	203 (31.7%)	75 (34.3%)	65 (32.3%)	38 (25.7%)	25 (34.3%)		65 (32.3%)	138 (31.4%)	
Involved Lymph node						0.12			0.087
• < 2	325 (42.9%)	105 (48%)	86 (42.8%)	59 (39.9%)	25 (34.2%)		86 (42.8%)	239 (54.3%)	
• ≥ 2	166 (25.9%)	49 (22.3%)	54 (26.8%)	39 (26.3%)	24 (32.9%)		54 (26.8%)	112 (25.5%)	
• Unknown & missing	200 (31.2%)	65 (29.7%)	61 (30.4%)	50 (33.8%)	24 (32.9%)		61 (30.4%)	89 (20.2%)	
Differentiation grade						0.054			0.049
• Well	114 (17.8%)	40 (18.2%)	30 (14.9%)	34 (23%)	10 (13.7%)		30 (14.9%)	84 (19.1%)	
• Moderate	318 (49.6%)	110 (50.2%)	10 4 (51.7%)	69 (46.6%)	35 (47.9%)		104 (51.7%)	214 (48.6%)	
• Poor	128 (20%)	42 (19.2%)	50 (24.9%)	28 (18.9%)	8 (11%)		50 (24.9%)	78 (17.79%)	
• Unknown & missing	81 (12.6%)	27 (12.4%)	17 (8.5%)	17 (11.5%)	20 (27.4%)		17 (8.5%)	64 (14.6%)	
Serum AFP level						0.095			0.068
• < 400 ng/ml	338 (52.7%)	111 (50.7%)	99 (49.3%)	80 (54.1%)	43 (58.9%)		99 (49.3%)	239 (54.3%)	
• ≥ 400 ng/ml	205 (32%)	75 (34.2%)	68 (33.8%)	50 (33.8%)	12 (16.4%)		68 (33.8%)	137 (31.1%)	
• Unknown & missing	98 (15.3%)	33 (15.1%)	34 (16.9%)	13 (12.1%)	18 (24.7%)		34 (16.9%)	65 (14.6%)	
Cirrhosis						0.11			0.098
• Yes	258 (40.2%)	90 (41.1%)	85 (42.3%)	67 (45.3%)	16 (21.9%)		85 (42.3%)	173 9.3%)	
• No	135 (21.1%)	48 (21.9%)	42 (20.9%)	28 (18.9%)	17 (23.3%)		42 (20.9%)	93 (21.1%)	
• Unknown & missing	248 (38.7%)	81 (37%)	74 (36.8%)	53 (35.8%)	40 (54.8%)		74 (36.8%)	174 (39.6%)	
Hepatitis type						0.084			0.14
• None	135 (21.1%)	42 (19.2%)	42 (20.9%)	32 (21.6%)	19 (26.1%)		42 (20.9%)	93 (21.1%)	
• HBV	314 (49%)	96 (43.8%)	101 (50.2%)	76 (51.3%)	41 (56.2%)		101 (50.2%)	213 (48.4%)	
• HCV	119 (18.6%)	50 (22.8%)	38 (18.9%)	28 (18.9%)	3 (4.1%)		38 (18.9%)	81 (18.4%)	
• HBV + HCV	17 (2.7%)	5 (2.3%)	3 (1.5%)	2 (1.4%)	7 (9.5%)		3 (1.5%)	14 (3.2%)	
• Unknown & missing	56 (8.6%)	26 (11.9%)	17 (8.5%)	10 (6.8%)	3 (4.1%)		17 (8.5%)	39 (8.9%)	
**RH**						0.58			0.66
• +	570 (88.9%)	197 (90%)	176 (87.6%)	132 (90.2%)	65 (89%)		176 (87.6%)	394 (89.5%)	
• -	71 (11.1%)	22 (10%)	25 (14.4%)	16 (10.8%)	8 (11%)		25 (14.4%)	46 (10.5%)	

#### Five-year survival rate

**Fig. 1:**
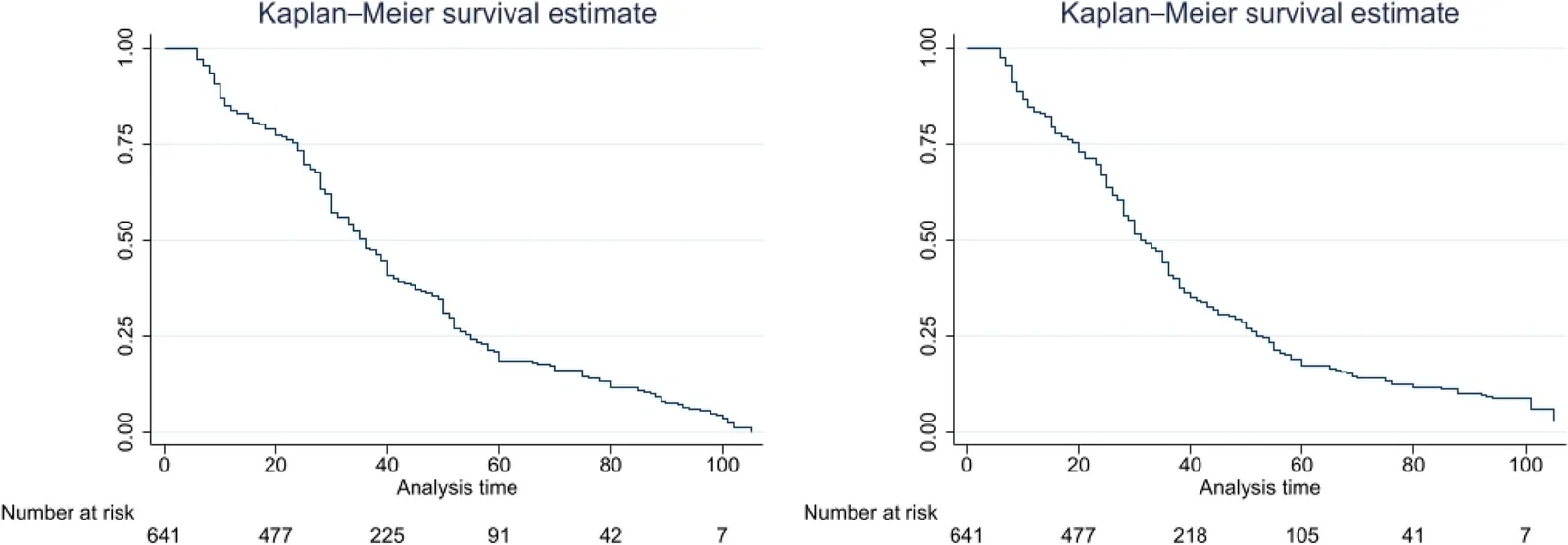
Kaplan Meier of 1, 3, and five OS and RFS rate

**Fig. 2:**
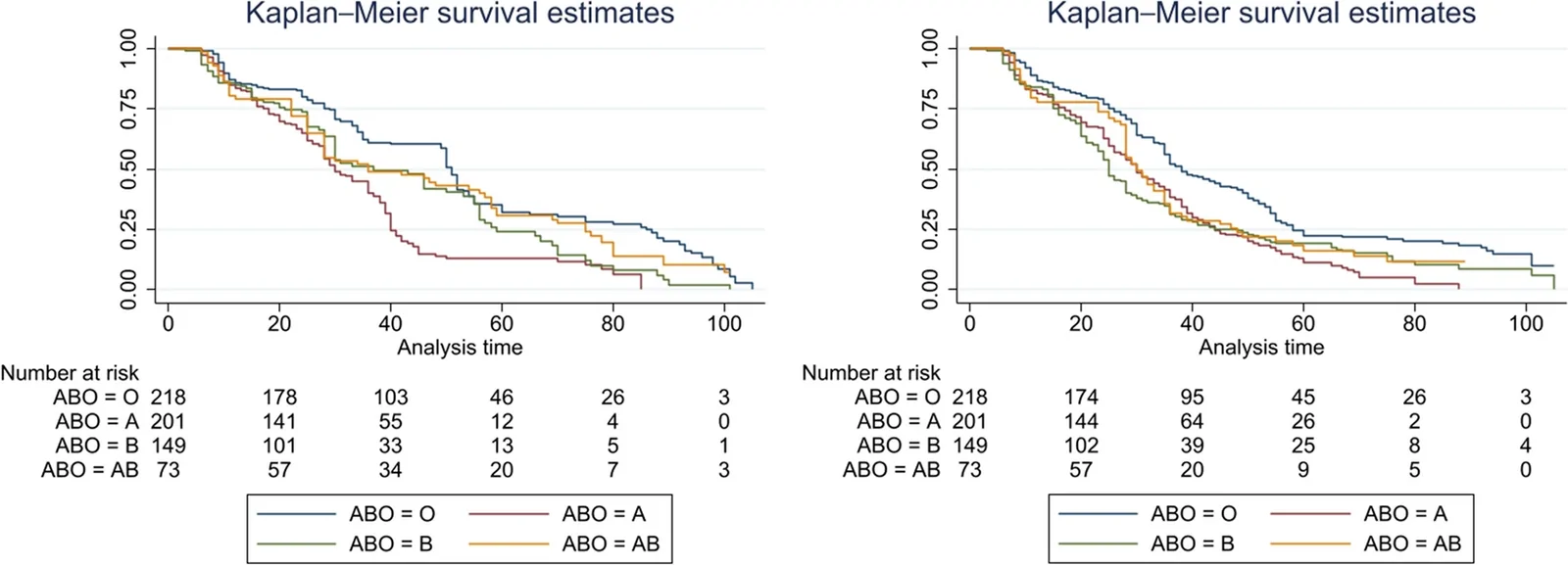
Kaplan–Meier of 5-year RFS and OS rate of patients based on blood type

**Fig. 3:**
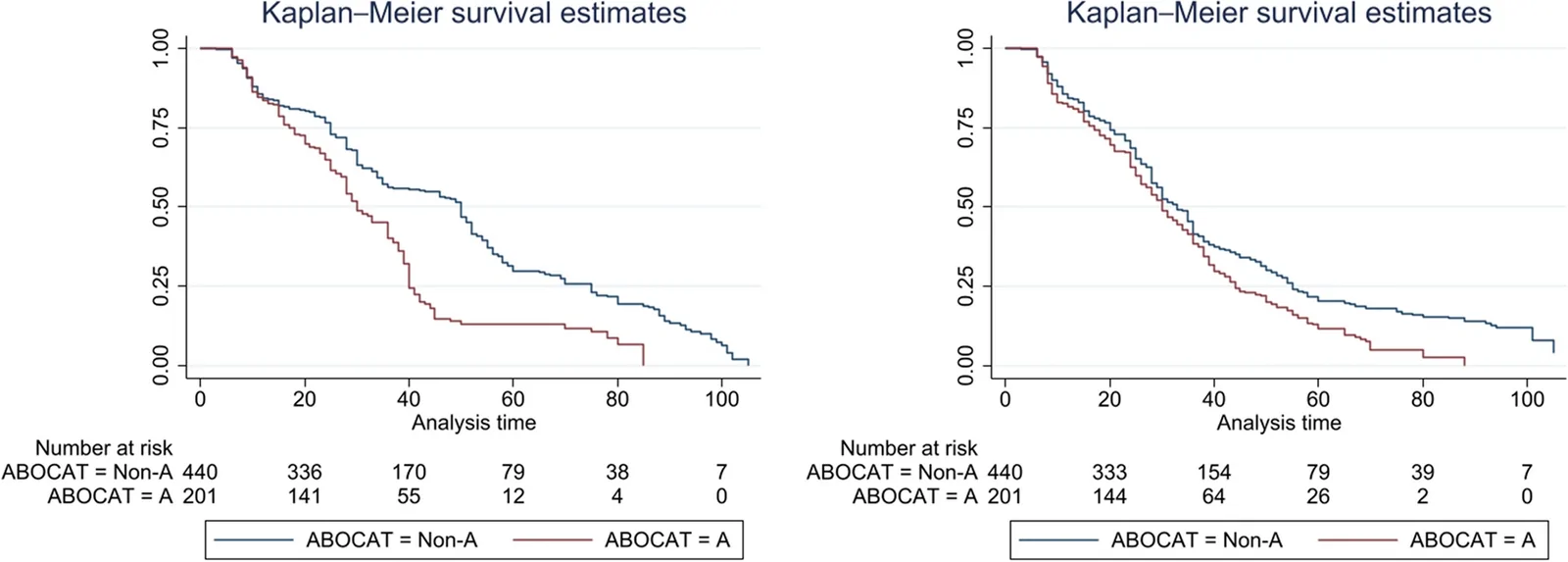
Kaplan-Meier of 5-year RFS and OS rate of patients based on blood type A versus non-A

The median OS and RFS time was 37 and 31 months, respectively. The five-year survival was 25.4%. The RFS rate was 18.7% (Fig. 1). The RFS rate for patients with blood groups A, O, B, and AB was 11.4%, 24.5%, 20.9%, and 19.9%, respectively. The highest and lowest 5-year survival rates were for patients with blood groups O and A, respectively. The OS rate for patients with blood groups A, O, B, and AB was 12.9%, 31.1%, 24.1, and 29.2%, respectively (Fig. 2). The OS rate in patients with blood type A was significantly lower than in non-A patients (12.9% vs. 29.7%). (Log rank: 40.89, P: 0.001) The five-year RFS rate in patients with blood group A was significantly lower than that of non-A blood groups (21.5% vs. 11.4%). (Log rank: 10.8, P: 0.001) (Fig. 3).

#### Univariate analysis

Based on the results of univariate analysis, the survival rate in patients aged 45 to 65 years was significantly better than in patients < 45 years old. (HR: 0.66, 95% CI: 0.59, 0.73, P:0.001) The 5-year survival rate is significantly related to tumor size, AFP level, the number of involved lymph nodes, differentiation grade, tumor size, hepatitis, blood group type, and metastasis were related. There was no significant association between Rh with OS and RFS (*P* > 0.05) (*P* < 0.05) Table 2.

**Table 2:** Predictors of 5-year survival of patients with HCC based on univariate analysis

Demographic characteristics	Five-year survival rate	HR (95% CI)	***P* value**
Age (year)			
• < 45	21.2%	Ref	-
• 45–65	30.7%	0.66 (0.59,0.73)	0.001
• > 65	21.4%	0.99 (0.55, 2.84)	0.81
BMI (Kg/m^2^)			
• ≤ 18	18.4%	Ref	-
• 18–25	29.8%	0.71 (0.60,0.83)	0.001
• > 25	23.2%	0.96 (0.71, 1.15)	0.087
Sex			0.25
• Male	24.9%	-	
• Female	26.9%		
Marital status			0.54
• Unmarried	27.2%	-	
• Married	25.2%		
Smoking status			0.61
• Yes	24.8%	-	
• NO	26.1%		
Education			
• Illiterate	25.3%	-	0.81
• < Diploma	24.2%		
• > = Diploma	26.1%		
Family history of GC			0.12
• No	27.2%		
• Yes	25.2%		
Alcohol consumption			0.44
• No	26.6		
• Yes	24.8		
• Unknown & missing	25.4		
Child–Pugh stage			0.12
• A	28.1	-	
• B	24.7		
• Unknown & missing	26.2		
Tumor size (cm)			
• ≤ 5	32.5%	Ref	-
• > 5	18.9%	1.73 (1.12,2.25)	0.001
• Unknown & missing	21.1%	1.48 (0.99,2.11)	0.074
Metastasis			
• No	31.6%	Ref	-
• Yes	21.2%	1.52 (1.11, 1.93)	0.001
• Unknown & missing	23.8%	1.32 (1.06,1.6)	0.004
Involved Lymph node			
• < 2	32.9%	Ref	-
• ≥ 2	20.11%	1.65 (1.16,2.17)	0.001
• Unknown & missing	24.6%	1.31 (1.04,1.62)	0.026
Differentiation grade			
• Well	33.2%	Ref	-
• Moderate	24.3%	1.36 (1.08,1.65)	0.006
• Poor	17.2%	1.94 (1.44,2.46)	0.0001
• Unknown & missing	25.1%	1.30 (1.01,1.62)	0.043
Serum AFP level			
• < 400 ng/ml	30.1%	Ref	-
• ≥ 400 ng/ml	20.4%	1.51 (1.12, 1.93)	0.0001
• Unknown & missing	25.6%	1.17 (0.96,1.43)	0.098
Cirrhosis			0.24
• Yes	26.6%	-	
• No	24.7%		
• Unknown & missing	25.3%		
ABO Blood Type			
• O	31.1%	Ref	-
• A	12.9%	2.41 (1.66,3.17)	0.001
• B	24.1%	1.29 (1.02,1.56)	0.045
• AB	29.2%	1.06 (0.86, 1.121)	0.18
ABO Blood Type			0.001
• Non-A	29.7%	Ref	
• A	12.9%	2.3 (1.46,3.15)	
Hepatitis type			
• None	28.3%	Ref	-
• HBV	20.14%	1.41 (1.02,1.81)	0.008
• HCV	25.1%	1.13 (0.97, 1.3)	0.084
• HBV + HCV	19.1%	1.42 (0.98,1.89)	0.092
• Unknown & missing	22.4%	1.25 (0.92, 1.59)	0.11
Hepatitis type			0.013
• Non-HBV	26.3%	Ref	
• HBV	20.14%	1.29 (1.04,1.55)	
RH		-	0.88
• +	25.8%		
• -	25.3%		

#### Multivariate analysis

To determine the effect of blood group type on survival adjusted for other tumor and demographic characteristics, all variables that had *p* < 0.2 in the univariate analysis were entered into the Cox multivariate analysis adjusted by the backward method. The multivariate analysis showed that the blood types A, age < 45 years, tumor size > 5 cm, poor tumor differentiation, presence of metastasis, number of lymph nodes ≤ 2, and serum AFP level ≥ 400 were associated with decreased survival of patients after hepatectomy (*P* < 0.05) Table 3.

**Table 3:** Independent predictors for survival rate by multivariate analysis

Demographic characteristics	**HR **_**Adju**_	**95% CI**	***P* value**
Age (< 45 vs > 45 year)	1.39	1.05, 1.74	< 0.001
BMI (Kg/m^2^)			
• ≤ 18	1.26	1.07,1.45	< 0.001
• 18–25	Ref	-	-
• > 25	1.19	1.01, 1.37	0.017
Tumor size (≤ 5 vs > 5 cm)	1.65	1.09, 2.22	< 0.001
Metastasis ( Presence vs absence)	1.50	1.10, 1.91	0.006
Involved Lymph node (≥ 2 Vs < 2)	1.62	1.14,1.81	< 0.001
Differentiation grade			
• Well	Ref	-	-
• Moderate	1.35	1.05,1.66	0.009
• Poor	1.90	1.41,2.39	< 0.0001
Serum AFP level (≥ 400 Vs < 400 ng/ml)	1.49	1.09,1.9	< 0.001
ABO Blood Type			
• O	Ref	-	-
• A	1.69	1.21,2.18	< 0.001
• B	1.19	0.98,1.41	0.11
• AB	1.02	0.88, 1.17	0.25
ABO Blood Type (A Vs. Non A)	1.65	1.15,2.16	< 0.001

*HR Adju* Hazard Ratio adjusted, *95% CI* 95% confidence interval

## Discussion

Although many studies have investigated the association between the ABO blood group type and the risk of HCC, limited studies have investigated the association between the ABO blood group type and the survival rate of HCC patients after hepatectomy or liver transplantation. Based on our knowledge, no comprehensive study has investigated the association between ABO blood group type with survival and RFS rate in Iranian patients with HCC after hepatectomy. Therefore, according to the issue’s importance, in this retrospective cohort study, we evaluated the effect of ABO blood group type and Rh of patients with OS and RFS rate after hepatectomy in a multicenter manner in 639 HCC patients.

According to the results of our study, the five-year OS and RFS rates of patients were 25.4% and 18.7%, respectively, indicating the disease’s lethality even after surgery. The highest and lowest survival and RFS rates were related to blood groups O and A, respectively. The 5-year RFS and OS rate in patients with blood group O was 24.5% and 31.1%, respectively, and for blood group A was 11.4% and 12.9%, which indicated the lower survival of patients with blood group A. In line with the results of our study, in 2022, M Kaibori et al. [22] showed that 5-year RF and OS rates after hepatectomy were the lowest in patients with blood type A. In addition, the results of our study showed that patients with blood type O had higher 5-year OS and RFS rates after hepatectomy. Based on the results of multivariate analysis, the 5-year survival rate in patients with blood group A compared to non-A, patients < 45 years old compared to patients ≥ 45 years old, patients with tumor size > 5 cm compared to < 5 cm, poor differentiation, the presence of metastasis before surgery, the number of lymph nodes ≤ 2, and the serum AFP level ≥ 400 was significantly lower, which was consistent with the results of studies conducted in this field [26, 31, 32]. T Wu et al., [21] by evaluating the association ABO blood group type with the survival rate of HCC patients after hepatectomy in a retrospective cohort study on 691 patients, showed that the frequency of blood types A and O were 28.8% and 37.9%, respectively. In line with the results of their study, in our study, the frequency of blood types O and A was 34.1% and 31.3%, respectively. They reported that patients with blood type O had the highest survival and patients with blood type A had the lowest survival rate, with a median survival of 39 months, which was consistent with the results of our study. In their study, they reported that in addition to the type of blood groups, tumor size > 5 cm, higher tumor stage, presence of metastasis, age < 45 years, poor tumor differentiation, and serum AFP level ≥ 400 ng/ml were significantly associated with to the decrease in the survival rate of patients after hepatectomy. In our study, in addition to these factors, a BMI ≤ 18 kg/m2 was associated with a decrease in patient survival, which can be justified due to anorexia, weakness, and poor health status of underweight patients, weakening the immune system. Several previous studies showed that BMI ≤ 18 was associated with decreased survival rates for patients with gastric cancer after surgery. Therefore, post-surgery patients’ BMI can be considered an essential independent prognostic factor [33, 34].

In another study in 2022, K Mohkam et al. [32], by examining the effect of ABO blood group type on liver cancer recurrence after liver transplantation on 925 patients with HCC, showed that the 5-year survival and recurrence rate in patients with Blood type A was significantly higher than patients with non-A blood type, which was consistent with the results of our study. In another study, K Bannangkoon et al. [35] evaluated the effect of ABO blood group type on the survival of patients with HCC treated with embolization. They showed that the patients with blood type O compared to non-O had a significantly higher survival rate, confirming our study’s results. Similar to the results of our study, Q Li et al. [29] showed that in patients with inoperable HCC after transarterial chemoembolization (TACE), the survival rate of patients with blood type O was higher than that of non-O. Based on the results of previous studies, the increased risk of cancer and the lower survival rate of patients with type A blood can be justified due to the reduced ability of the immune system of patients with type A blood to identify and attack tumor cells expressing antigens that are structurally similar to ABO antigen [36, 37]. Contrary to the results of our study, A Oral et al., [27], by examining 502 cirrhotic patients with HCC, did not report a significant relationship between the ABO blood group with survival rate. This difference can be justified due to the difference in the characteristics of the patients under investigation and the difference in the sample size in the two studies. Their study examined only cirrhotic patients, while both cirrhotic and non-cirrhotic patients were examined in our study. Another strength of our study compared to this study was the larger sample size, conducting the study in a multicenter manner, and examining more factors adjusted for other factors.

Our study had limitations and strengths that should be noted. The retrospective study design was the most important weakness of our study, which made us unable to measure several important factors such as comorbidities, R status of resection, adjuvant chemotherapy or portal vein embolization before surgery (to assess for increased residual liver function) and tumor markers. In addition, due to the retrospective nature (Prone to selection bias) and the use of patients’ medical profiles, there were missing cases in a number of variables, which can affect the estimation of the results. Our study was conducted on patients with specific characteristics, so the results should be generalized to other populations with caution. The design of prospective studies with a large sample size is recommended to estimate the results more accurately. Designing a comprehensive multicenter study with a high sample size, for the first time on the Iranian population, to evaluate the effect of ABO blood group type on the survival rate of patients with HCC in conditions adjusted for other variables was one of the most important strengths of our study.

## Conclusion

Our study showed that the blood group type as an independent factor was related to the five-year OS and RFS rate in patients with HCC after hepatectomy. Compared to non-A, blood type A can be associated with decreased five-year OS and RFS rates in patients with HCC after surgery. The five-year OS and RFS rates were higher in patients with blood type O than non-O. Prospective studies are recommended to estimate the results more accurately.

## Data Availability

The datasets generated or analyzed during the current study are available from the corresponding author on reasonable request.

## Abbreviations

HCC: Hepatocellular carcinoma
OS: Overall survival
RFS: Recurrence-free survival
AFP: Alpha-Fetoprotein
BMI: Body mass index
TNM: Tumor-node-metastasis index
HR: Hazard Ratio
95% CI: 95% Confidence interval
