# Correction: Effect of ABO blood group on postoperative overall survival and recurrence-free survival rate in patients with hepatocellular carcinoma after hepatectomy: a multi-center retrospective cohort study

**DOI:** 10.1186/s12893-026-04041-5

**Published:** 2026-08-28

**Authors:** Mansour Bahardoust, Maryam Zolfaghari Dehkharghani, Pouya Ebrahimi, Maryam Najafirashed, Safa Mousavi, Meisam Haghmoradi, Mohsen Khaleghian, Adnan Tizmaghz

**Affiliations:** 1 Department of Epidemiology, School of Public Health, Shahid Beheshti University of Medical Sciences, Tehran, Iran; 2 Department of Health Care Administration and Policy, School of Public Health, University of Nevada Las Vegas(UNLV), Las Vegas, NV USA; 3 Ahvaz Jundishapur University of Medical Sciences, Ahvaz, Iran; 4 School of Medicine, Iran University of Medical Sciences, Tehran, Iran; 5 Department of Public Health, College of Health and Human Services, California State University, Fresno, CA USA; 6 Department of Orthopedic Surgery, Urmia University of Medical Sciences, Urmia, Iran; 7 Vascular Surgery Department of General Surgery, School of Medicine, Iran University of Medical Sciences, Tehran, Iran; 8 Department of General Surgery, School of Medicine, Iran University of Medical Sciences (IUMS), Tehran, Iran

**Correction to: BMC Surg (2023) 23:324**

**https://doi.org/10.1186/s12893-023-02236-8**

After publication, and following post-publication peer review by the Journal, several issues were identified in the published article [1]. The authors are therefore making the following corrections to the Patient Characteristics and Discussion sections to address the identified issues:

On page 2, paragraph 6, “underwent resection hepatectomy” was added after “the medical profiles of 956 patients with HCC who”, and “patients who underwent lobectomy” was added after “secondary HCC (cases that did not originate from the liver)”.

On page 7, paragraph 18, “Comorbidities, R status of resection, adjuvant chemotherapy or portal vein embolization before surgery (to assess for increased residual liver function)” was added after “which made us unable to measure several important factors”. “(Prone to selection bias)” was added after “in addition, due to the retrospective nature”. “Our study was conducted on patients with specific characteristics, so the results should be generalized to other populations with caution” was added after “there were missing cases in a number of variables, which can affect the estimation of the results.”

The original article has been corrected.
